# Screen-Printed Li Arrays with Reinforced Interphase as Ultrathin Anodes for High-Energy–Density Liquid- and Solid-State Batteries

**DOI:** 10.1007/s40820-026-02342-1

**Published:** 2026-08-21

**Authors:** Wenting Liu, Zhenyao Wei, De Ning, Zhenhuan Lu, Fengping Zhang, Xuhao Wang, Binzhe Zhao, Guohua Zhong, Jie Zhang, Yan Shao, Ming Chen, Man Wang, Jun Wang, Chunlei Yang, Wei Wu

**Affiliations:** 1 Guangxi Key Laboratory of Electrochemical and Magneto-chemical Functional Materials, College of Chemistry and Bioengineering, Guilin University of Technology, Guilin, 541004 People’s Republic of China; 2 Shenzhen Institutes of Advanced Technology, Chinese Academy of Sciences, Shenzhen, 518055 People’s Republic of China; 3 Department of Materials Science and Engineering, School of Innovation and Entrepreneurship, Southern University of Science and Technology, Shenzhen, 518055 People’s Republic of China; 4 Faculty of Materials Science and Energy Engineering, Shenzhen University of Advanced Technology, Shenzhen, 518107 People’s Republic of China

**Keywords:** Lithium metal battery, Ultrathin anode, Array, Energy density, Solid-state battery

## Abstract

High-energy–density lithium metal batteries, either liquid or solid state, require ultrathin Li anodes, but their implementation is hindered by poor Li processability in fabrication and inhomogeneous Li plating behaviours upon cycling. This work addresses these challenges through a mask-patterned discrete array using a molten salt-derived Li nanocomposite. The screen-printing strategy circumvents the poor wettability of molten Li on copper that causes high Li|Cu contact angle and non-uniform Li spreading, thus fabricating ultrathin Li foils with equivalent thicknesses below 30 μm. Concurrently, the molten salt-derived inorganic nanoparticles in Li favour homogeneous Li deposition and induce inorganic-rich solid electrolyte interphase to jointly suppress active Li consumption. The resulting Li array (10–28 μm) anodes demonstrate remarkable performance in both liquid- and solid-state systems, achieving high energy densities up to 504 Wh kg^−1^/1071 Wh L^−1^ and stable cycling over 260 cycles with high-loading cathodes. This integrated approach resolves both fabrication and cycling challenges of ultrathin Li anodes and offers an alternative solution for high-performance liquid-/solid-state batteries.

**Supplementary Information:**

The online version contains supplementary material available at 10.1007/s40820-026-02342-1.

## Highlights

- A screen-printing strategy using mask-patterned discrete arrays overcomes molten Li’s poor wettability on Cu, enabling fabrication of ultrathin Li anodes (< 30 μm).
- In situ generated inorganic nanoparticles from Mg(TFSI)_2_ homogenize Li deposition and induce an inorganic-rich solid electrolyte interphases, significantly suppressing active Li consumption.
- The Li@Mg(TFSI)_2_ array anode fits both liquid- and solid-state full cells, achieving high energy densities of 504 Wh kg^−1^/1071 Wh L^−1^ and stable cycling over 260 cycles.

**Supplementary Information:**

The online version contains supplementary material available at 10.1007/s40820-026-02342-1.

## Graphical abstract

**Figure:**
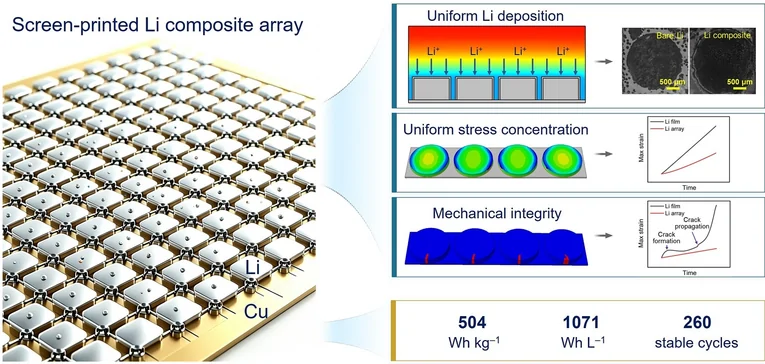

## Introduction

Lithium metal batteries (LMBs) represent the pinnacle of high-energy–density storage systems, owing to Li’s unparalleled theoretical capacity (3860 mAh g^−1^) and the lowest electrochemical potential (− 3.04 V vs. SHE) [1]. These intrinsic advantages position LMBs as the most promising candidates to meet the escalating energy demands of next-generation applications, particularly in aerospace, electric vehicles, and portable electronics [2]. However, the practical deployment of LMBs has been hindered by the challenges associated with conventional thick Li foils (≥ 50 μm), including excessive inactive Li mass (15%–25% of total cell weight), accelerated dendrite growth, and severe volume fluctuations during cycling [3]. Ultrathin Li foils (≤ 20 μm) emerge as a transformative solution, dramatically reducing inactive Li while enhancing energy density (e.g., achieving > 400 Wh kg^−1^ and > 1000 Wh L^−1^) and cycling stability [4]. Their adoption is indispensable for unlocking the full potential of LMBs, yet scalable fabrication of such foils remains a critical bottleneck [3].

Existing approaches to ultrathin Li production include electrochemical deposition, physical vapour deposition (PVD), and roll pressing [4]. Electrochemical deposition enables precise thickness control (down to micron level) but suffers from low throughput and electrolyte consumption [5]. PVD and the thermal evaporation pathway achieve submicron uniformity yet need ultrahigh vacuum and incur prohibitive costs [6]. Roll pressing struggles to produce foils below 20 μm without cracking or heterogeneity [7]. In contrast, molten Li coating, combining scalability, cost-effectiveness, and thickness tunability, stands out as the most viable industrial-scale solution [7]. However, the practical realization of molten Li coating is severely constrained by the poor wettability of Li on copper substrate, manifested by high contact angles (> 90°) and inhomogeneous spreading [8]. This arises from the high surface tension of molten Li, which hampers the Li spreading away on copper [9]. Achieving uniform, thin, and large-area Li coating without defects (cracks, pinholes) remains elusive, limiting LMBs’ industrial adoption. Addressing this requires innovative strategies to modulate both the surface energy of Li and the interfacial energy of Li|Cu interface, such as lithiophilic nanocoating or alloying, to enable a spontaneous Li spreading [8]. The development of such approaches is urgent to bridge the gap between laboratory-scale breakthroughs and the mass production of high-performance ultrathin Li anodes for next-generation batteries.

Continuous consumption of active Li during cycling is another persistent challenge that limits the practical implementation of Li anodes [10]. This irreversible loss originates from multiple parasitic reactions between Li metal and electrolytes, leading to the formation of unstable solid electrolyte interphases (SEIs) and dead lithium [11]. Repeated Li plating/stripping processes further exacerbate this issue through dendrite growth and electrolyte decomposition [1]. The problem becomes particularly severe in ultrathin Li anodes (≤ 20 μm), where the limited Li inventory makes every loss of active Li directly detrimental to cycling performance [12]. As cycling progresses, the accumulated Li loss causes rapid capacity fading and severely limits battery lifespan [13]. This fundamental problem persists across both liquid- and solid-state battery systems, becoming a critical obstacle. To address this challenge, modifying Li composition has emerged as an essential strategy [14]. By introducing functional additives or coatings, the Li surface chemistry can be engineered to form more stable interfaces and regulate deposition behaviours [15]. Such compositional modifications offer a promising pathway to mitigate active Li consumption and achieve long-term cycling stability in practical LMBs.

Herein, a dual-modulation approach is proposed to confront the issues on both Li fabrication and cell cycling. On the one hand, a mask-patterning technique is developed to circumvent the wettability challenge by producing uniform, discrete Li arrays (equivalent 10–28 μm thick) instead of films, solving the fabrication bottleneck. On the other hand, the in situ-generated inorganic nanoparticles by the reactions between magnesium bis(trifluoromethanesulfonyl)imide (Mg(TFSI)_2_) and molten Li favour a homogeneous Li deposition and induce an inorganic-rich SEI to jointly minimize parasitic reactions and active Li consumption. Synergizing these innovations, ultrathin Li@Mg(TFSI)_2_ arrays achieve energy densities exceeding 504 Wh kg^−1^/1071 Wh L^−1^ in full cells with high-loading cathodes. Li@Mg(TFSI)_2_ arrays are versatile in both liquid- and solid-state cells, presenting the elusive balance of high energy density, long cycle life, and structural stability. This work resolves both the fabrication and cycling limitations of ultrathin Li anodes, providing a scalable path towards commercial high-energy–density LMBs.

## Experimental Section

### Materials

Mg(TFSI)_2_ (99.9%) and liquid electrolytes (1.0 M lithium bis(trifluoromethanesulphonyl)imide (LiTFSI) in 1,3-dioxolane (DOL):dimethoxy ethane (DME) = 1:1 vol% with 2 wt% LiNO_3_; 1.0 M lithium hexafluorophosphate (LiPF_6_) in ethylene carbonate (EC):methyl (2,2,2-trifluoroethyl) carbonate (FEMC) = 3:7 (vol%)) were purchased from Suzhou Duoduo Chemical Technology Co., Ltd. Polyethylene glycol diacrylate (PEGDA) (Mn = 1000), trifluoroethyl acrylate (TFEA), 2,2,3,4,4,4-hexafluorobutyl acrylate (HFBA), and 2,2′-azobis(2-methylpropionitrile) (AIBN) were purchased from Aladdin Co., Ltd. High-loading LFP cathodes of 12.3 mg cm^−2^ (≈ 2.0 mAh cm^−2^) and 20.0 mg cm^–2^ (≈ 3.3 mAh cm^−2^), LCO cathode of 12.0 mg cm^−2^ (≈ 2.0 mAh cm^−2^), NCM811 cathodes of 9.2 mg cm^–2^ (≈ 1.9 mAh cm^−2^), 16.9 mg cm^–2^ (≈ 3.4 mAh cm^−2^), and Ni92 of 22.9 mg cm^–2^ (≈ 4.6 mAh cm^−2^) were obtained from Guangdong Canrd New Energy Technology Co., Ltd.

### Preparation of Thin Anode Foils

Stainless steel masks with designed dimensions and periodic circular apertures were fabricated using a femtosecond laser. Li metal and Mg(TFSI)_2_ powder were heated to a molten state in a stainless steel crucible. Then, the molten mixture was poured and cast onto a mask that placed atop Cu foil using an applicator roll. Molten Li@Mg(TFSI)_2_ was forced to fill the openings fluidly, and a regular array patterning with isolated Li microdiscs would be constructed upon mask removal. The prepared foil was termed as Li@Mg(TFSI)_2_ and was used for the subsequent characterizations and electrochemical measurements. Bare Li foil was similarly prepared. All the experiment steps were conducted in an argon-filled glove box (H_2_O ≤ 0.01 ppm, O_2_ ≤ 0.01 ppm).

### Physical Characterizations

Both the surface and cross-sectional morphologies of bare Li and Li@Mg(TFSI)_2_ were imaged using a SEM (Hitachi S-4800). The surface composition was analysed by XPS (Escalab 250Xi). The contact angle of molten Li on Cu substrate under different temperatures (190–300 °C) was measured using a high-temperature high-vacuum contact angle goniometer. It operates on the principle of optical imaging, measuring the contact angle on certain surface under elevated temperatures through image contour analysis. The system comprises six major components, light source, high-temperature system, vacuum system, image acquisition, cooling system, and analysis software. The goniometer was operated in argon atmosphere. The internal microstructure of Li@Mg(TFSI)_2_ was characterized using coupled focused ion beam and scanning electron microscopy (FIB-SEM) (Thermo Scientific Helios 5 CX) at an accelerating voltage of 5 kV.

### Electrochemical Measurements

Li||Li symmetric cells, Li||LFP, Li||LCO, Li||NCM811, and Li||Ni92 cells in the configuration of CR2032 were assembled to explore the cell performance of both bare Li and Li@Mg(TFSI)_2_. For liquid cells, electrolyte (1.0 M LiTFSI in DOL: DME = 1:1 vol% with 2 wt% LiNO_3_) was used. For solid cells, SSE layer was prepared by dissolving 1 wt% PEGDA crosslinker and the monomers TFEA and HFBA (1:1 wt%) in electrolyte (1.0 M LiPF_6_ in EC:FEMC = 3:7). After thorough stirring, 1 wt% AIBN (relative to the mass of the polymer monomers) was added, and the mixture was stirred evenly again to assemble the solid-state cells. The SSLMB cells were then placed in an oven at 60 °C for 10 h to achieve in situ polymerization and solidification.

Symmetric cells were cycled at a current density of 1 mA cm^−2^ with an areal capacity of 1 mAh cm^−2^. The cut-off voltage ranges of Li||LFP, Li||LCO, Li||NCM811, and Li||Ni92 cells were set as 2.5–3.8 V for LFP and 3.0–4.3 V for LCO, NCM811, and Ni92 using a Land CT2001A battery testing system. Ionic conductivity and LSV of SSE were measured with a Solartron multi-channel potentiostat electrochemical workstation (1260 A) at room temperature. The ionic conductivity was measured by electrochemical impedance spectroscopy (EIS) using the perturbation signal of 10 mV over the frequency of 1 MHz–10 Hz. The SSE was sandwiched with stainless steel (SS) blocking electrodes with SS|SSE|SS configuration. The electrochemical stable window of SSE was evaluated by LSV over the potential of 0–7.0 V with a scanning rate of 0.5 mV s^−1^, using Li|SSE|SS configuration. The Li-ion transference number was investigated with the combination of AC impedance and DC polarization, using asymmetrical blocking electrode of Li|SSE|Li configuration. AC impedance of symmetrical cells was detected over the frequency of 1 MHz–0.01 Hz by EIS. The voltage of DC polarization is 10 mV. The value of *t*^+^ was calculated by Bruce–Vincent–Evans Equation [16].

### Computational Method

The surface structures were simulated by constructing the slab model. The slab was separated by a 20 Å vacuum space in the z direction to avoid interaction between layers. The surface structures were built by cleaving bulk Li and Li compounds along (110) or (111) plane. The adsorbing modes were built by adsorbing Li atom on the surface structures. The structural optimization, total energy, and surface electronic states of these structural configurations were calculated by employing the Vienna ab initio simulation package (VASP) within the framework of density functional theory (DFT). In these calculations, the projector augmented wave method and the exchange–correlation functional of generalized gradient approximation (GGA) were adopted. The energy cut-off was set as 450 eV for the plane-wave basis-set expansion. The k-point of the Brillouin zone was 0.03 Å^−1^ interval distribution of Monkhorst–Pack for the optimization of structures, and the k-point interval of the total energy self-consistent calculation was 0.02 Å^−1^ or better. All the structures were fully optimized, and convergence thresholds were set as 10^−5^ eV in energy and 10^−3^ eV Å^−1^ in force. Based on the calculated total energies, the surface adsorption energy was estimated as *E*_ads_ = [*E*_(surface-adsorber)_–*E*_surface_–*E*_adsorber_]/*N*, where *E*_(surface-adsorber)_ and *E*_surface_ are the total energy of the adsorbed and bare slab, respectively, *E*_adsorber_ is the total energy of the adsorber, and *N* is the number of atoms in the slab.

### Simulation

COMSOL Multiphysics software with the implemented finite element solver was used to study the Li^+^ concentration and Li^+^ flux distribution near the electrode/electrolyte interface using different anodes. The electrolyte conductivity was 0.5 S m^−1^; lithium transference number was 0.5. Mg(TFSI)_2_-derived dopants were represented by a 0.5-μm-thick layer (Li^+^ diffusion coefficient 1 × 10^−10^ m^2^ S^−1^) on top of bare Li. Moreover, finite element analysis (FEA) was conducted using the ABAQUS software to simulate the electro-chemo-mechanical behaviour of traditional continuous Li film and the proposed Li array upon Li deposition. The material properties were defined as follows: for Li metal, a Young’s modulus of 4.9 GPa and a Poisson’s ratio of 0.36 were adopted; for the Cu substrate, the Young’s modulus and Poisson’s ratio were set to 120 GPa and 0.34, respectively [17]. The model geometry consisted of solid elements for the bulk Li and Cu components to accurately capture volumetric deformation, while three-dimensional planar elements were used to discretize the initial crack structure. Static analysis steps were employed to simulate the quasi-static stress distribution during Li electrodeposition, allowing for the gradual accumulation of mechanical stress. Crack propagation was modelled using the eXtended Finite Element Method (XFEM), which enables the accurate tracking of crack growth without re-meshing, thus capturing the evolution of crack morphology and stress concentration under mechanical loading.

## Results and Discussion

### Mass Fabrication of Thin Li Foils Impeded by Large Li|Cu Contact Angle

**Fig. 1:**
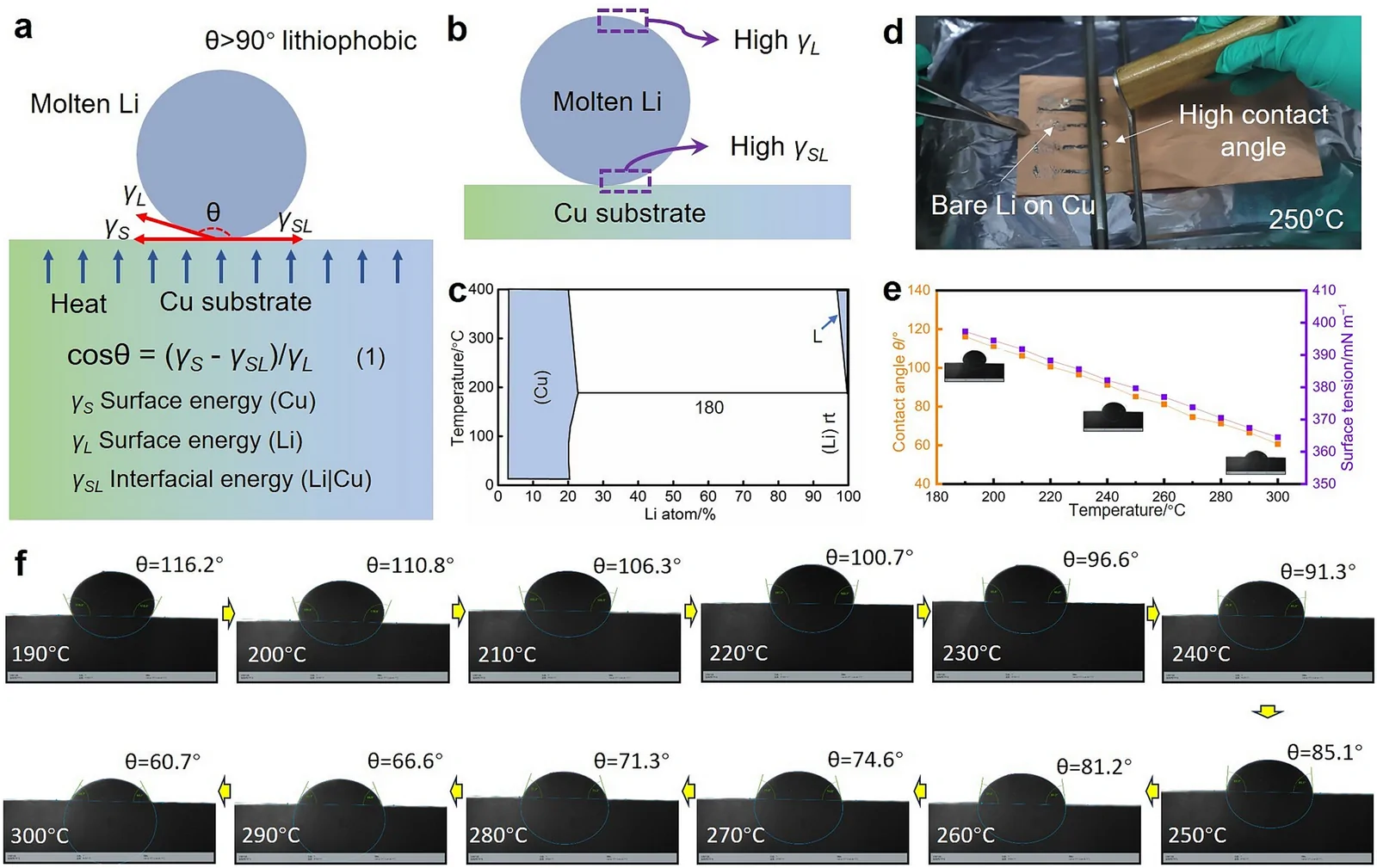
Schematics depicting the large Li|Cu contact angle. **a** Illustration of the equilibrium Young’s contact angle *θ* of molten Li on Cu. **b** High surface energy of Li *Y*_*L*_ and high interfacial energy of Li|Cu interface *Y*_SL_ jointly contribute to the large contact angle of Li|Cu. **c** Binary phase diagram of Li-Cu. **d** Snapshot of molten Li coating on Cu, showing the high contact angle at the Li|Cu interface. **e** Variations in both contact angle of Li|Cu and surface tension of molten Li as a function of temperature. **f** Observation of contact angle modulation of molten Li on Cu from 190 to 300 °C

Reprocessing molten Li metal represents a scalable meanwhile cost-effective approach to fabricate Li foils [8]. However, the ease of Li spreading away on Cu is heavily hampered by the bad Li wettability on Cu and a high Young’s contact angle *θ* (Eq. 1 in Fig. 1a). Molten liquid metals are usually high-surface energy liquids, reflecting the high cohesion of metals due to their metallic (i.e. chemical) bonding. Such bonding is generally one to two orders of magnitude greater than the surface energies of room temperature liquids by weak intermolecular interactions (i.e., physical interactions) [18]. Moreover, the lithiophobic nature of Cu towards molten Li also results in a high interfacial energy* Y*_SL_ of the Li|Cu interface. High surface energy* Y*_*L*_ of molten Li and high interfacial energy* Y*_SL_ of Li|Cu jointly contribute to a large contact angle of Li|Cu (Fig. 1b). Though the alloying reaction between molten Li and the heated Cu surface above 250 °C is proven to occur [19] as a small quantity of Li initiates to dissolve into Cu within the narrow soluble region (blue areas in Fig. 1c), the heat-triggered LiCu_*x*_ solid solution skeleton in the Li|Cu interface still cannot ensure a good enough wettability to coat thin Li layers in a flat and smooth manner (Figs. 1d and S1, Video S1). In Fig. 1e, f, molten Li exhibits temperature-sensitive wetting behaviour on Cu under argon atmosphere, where in situ measurements confirm *θ* remains > 90° (116.2° to 91.3°) across 190–240 °C, and reduces to 60.7° at 300 °C. The Li|Cu interface retains inherent lithiophobicity, with contact angles remaining unacceptably high for practical coating applications even at elevated temperatures.

### Quantitative Design of Ultrathin Li Foils in Form of Array as Alternatives

**Fig. 2:**
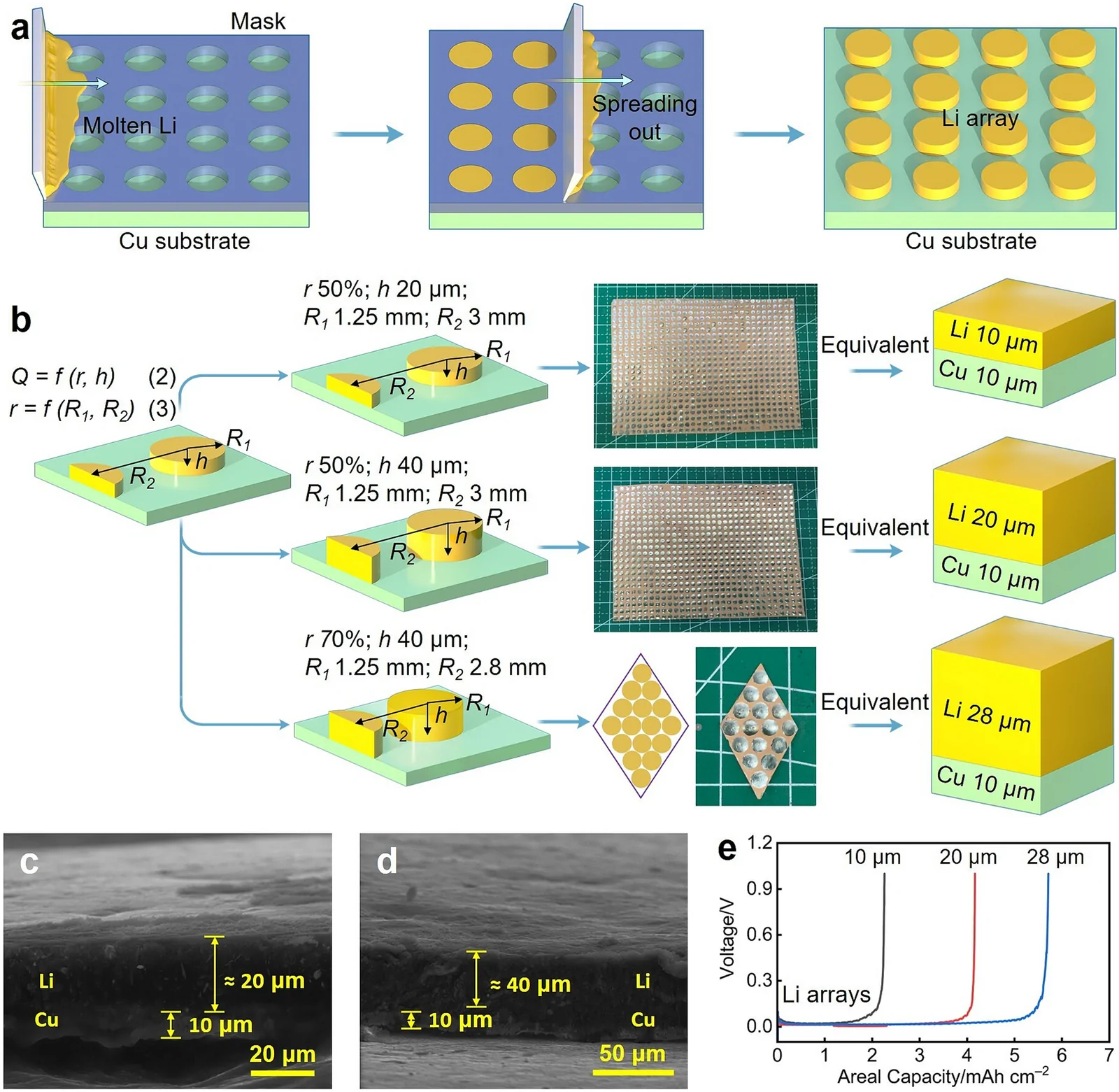
Mask-assisted fabrication of ultrathin-equivalent Li arrays to bypass lithiophobic Li|Cu wetting limitations. **a** Schematic illustration of the mask-assisted spreading process. Molten Li is blade-coated through a patterned mask, forming discrete Li arrays on Cu after mask removal. **b** Design principle for Li arrays, i.e. areal capacity (*Q*) depends on Li thickness (*h*) and coverage ratio (*r*), with optical images of the resulting 10/20/28 μm-equivalent Li anodes. **c** Cross-sectional SEM images confirming Li array thicknesses of c) ≈ 20 μm and **d** ≈ 40 μm on Cu foil. **e** Voltage curves of Li arrays with areal capacities of ≈ 2.0 mAh cm^−2^ (equivalent 10 μm), ≈ 4.1 mAh cm^−2^ (equivalent 20 μm) and ≈ 5.5 mAh cm^−2^ (equivalent 28 μm)

Building on the previous observation that planar molten Li coating is severely limited by the lithiophobic Cu surface, we herein propose a mask-assisted spreading strategy to bypass the intrinsic wetting barrier. Rather than attempting to achieve uniform continuous coating, inspired by semiconductor manufacturing, this approach leverages screen-printed patterning to fabricate discrete Li arrays, which collectively function as an ultrathin “effective anode” with tunable areal capacity. As schematically illustrated in Fig. 2a, a photolithographically defined mask with periodic circular apertures is placed atop Cu foil, and molten Li is blade coated through the openings. Upon mask removal, isolated Li microdiscs remain, forming a regular array patterning with precisely controlled thickness (*h*) and surface coverage ratio (*r*). This method avoids direct contact between molten Li and the entire Cu surface, mitigating wettability issues and enabling ultrathin-equivalent performance without requiring continuous coating.

To achieve tunable areal capacity, the equivalent Li areal capacity (*Q*) is mathematically defined as a function of the physical Li disc thickness (*h*) and the Li-to-Cu coverage ratio (*r*) via *Q* = *f*(*r*, *h*) (Eq. 2 in Fig. 2), where *r* depends on the mask geometry, i.e. individual Li dot radius (*R*_*1*_) and adjacent dot centre-to-centre distance (*R*_*2*_) (*r* = *f*(*R*_*1*_, *R*_*2*_)) (Eq. 3 in Fig. 2). For practical implementation, we fixed *r* = 50% (*R*_*1*_ = 1.25 mm, *R*_*2*_ = 3 mm) and tuned *h* to achieve two target capacities: ≈ 2 and ≈ 4 mAh cm^–2^, corresponding to “effective thicknesses” of 10 and 20 μm for continuous foils, respectively (Fig. 2b). Increasing *r* to 70% by reducing *R*_*2*_ further boosts equivalent thickness to 28 μm, demonstrating design flexibility. Cross-sectional scanning electron microscopy (SEM) images confirm the precise control over Li array dimensions. Figure 2c shows a ≈ 20-μm-thick Li disc on 10-μm Cu foil, while Fig. 2d verifies a ≈ 40-μm-thick array, matching the designed *h* values. Using mask template enables large-area fabrication up to ≈ 30 × 10 cm^2^ (Fig. S2). The discrete Li arrays from different batches exhibit uniform morphology without cracks, confirming good batch‑to‑batch reproducibility of the fabrication process. Moreover, both peel tests (Fig. S3) and bending tests (Fig. S4) were performed to evaluate the adhesion strength and mechanical robustness of the screen‑printed Li microdiscs on Cu. The peel test demonstrates excellent adhesion between Li microdiscs and Cu substrate, with no delamination observed. After 200 repeated bending, the individual Li microdiscs remain intact in morphology and maintain tight contact with Cu, without any visible physical damage or detachment. These results confirm the superior mechanical robustness of the Li array electrode.

The voltage curves shown in Fig. 2e further confirm the functional equivalence of the Li array to continuous ultrathin foil. The samples exhibit the characteristic Li plating/stripping plateau at ≈ 0 V vs. Li/Li^+^, with areal capacities of ≈ 2.0 mAh cm^−2^ (10 μm-equivalent), ≈ 4.1 mAh cm^−2^ (20 μm-equivalent), and ≈ 5.5 mAh cm^−2^ (28 μm-equivalent), closely matching theoretical values. The absence of voltage polarization anomalies indicates that the discrete array structure does not introduce extra interfacial resistance, validating its functionality as an anode. This design thus circumvents the poor interfacial affinity challenge by replacing the need for continuous spreading with a “patchwork” of lithiophilic-confined Li domains, offering a pragmatic and scalable pathway to ultrathin Li anodes. This mask-assisted strategy represents a transformative departure from conventional coating methods, i.e., by embracing the lithiophobicity instead of fighting it, thus achieving precise control over Li loading and thickness.

### Molten Salt-Derived Li Nanocomposite Engineering for Mask-Patterned Arrays

**Fig. 3:**
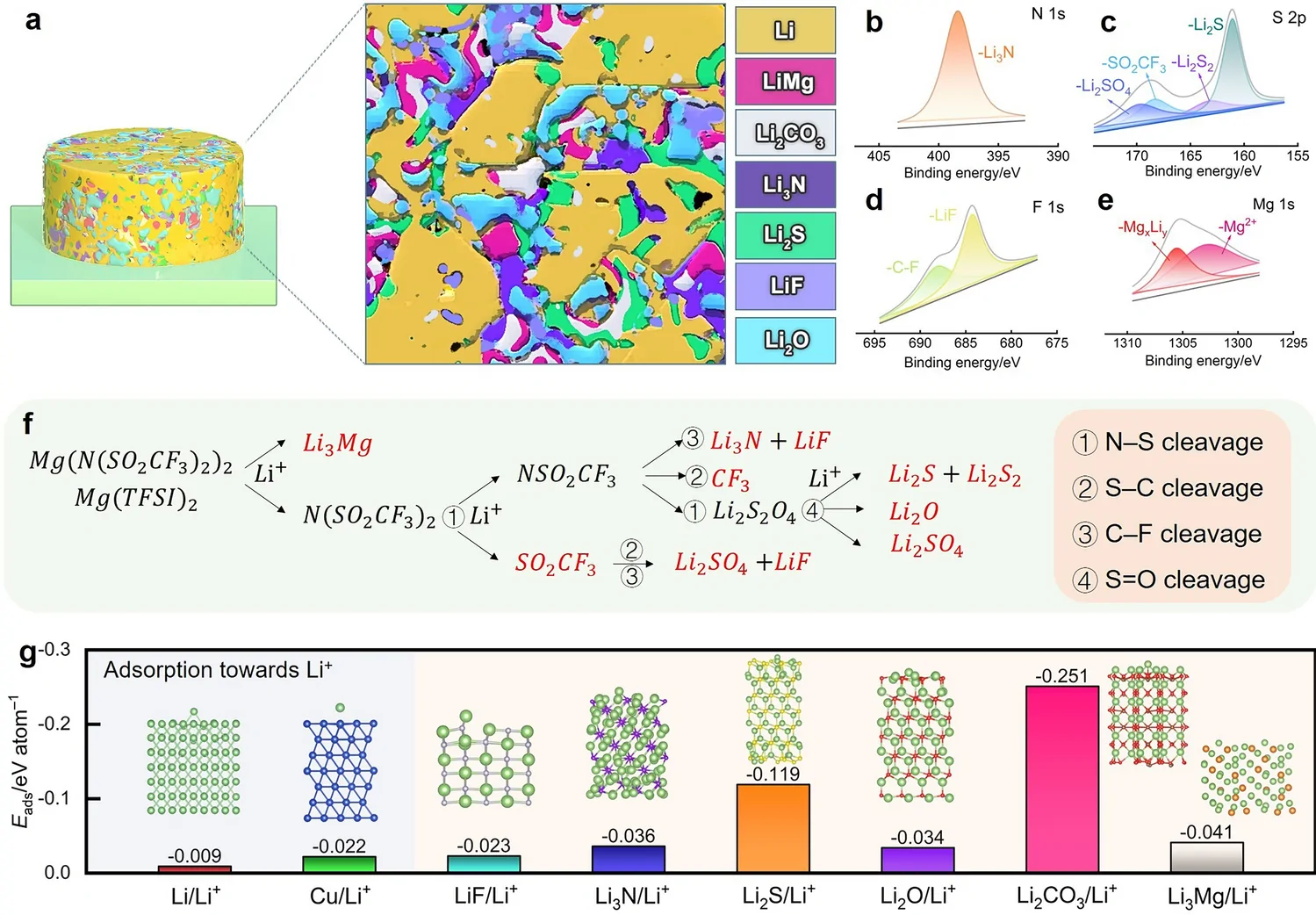
Mg(TFSI)_2_-driven in situ Li nanocomposite engineering for mask-patterned arrays, with component analysis and reaction pathways. **a** Schematic of Mg(TFSI)_2_-Li reactions to form a multi-component nanocomposite. XPS spectra of nanocomposite: **b** N 1*s* shows Li_3_N; **c** S 2*p* confirms Li_2_S/Li_2_S_2_/Li_2_SO_4_; **d** F 1*s* indicates LiF; **e** Mg 1*s* reveals Mg-Li alloy Li_3_Mg and oxidized Mg^2+^. **f** Reaction pathway of Mg(TFSI)_2_ decomposition. TFSI^−^ undergoes N–S cleavage, S–C cleavage, C–F cleavage and S=O cleavage, generating Li_3_N, LiF, Li_2_S and Li_2_O, etc. **g** *E*_ads_ values of Li^+^ on bare Li, Cu, LiF, Li_3_N, Li_2_S, Li_2_O, Li_2_CO_3_ and Li_3_Mg

To synergistically enhance the interfacial stability of mask-patterned Li arrays upon cycling, we herein introduce Mg(TFSI)_2_ doping into molten Li before casting, inducing in situ reactions between Mg(TFSI)_2_ and Li to form a multi-component nanocomposite (Fig. 3a). This strategy not only inherits the geometric control of discrete Li arrays but further modulates the surface chemistry, addressing the intrinsic lithiophobicity and poor SEI stability of bare Li. Mg(TFSI)_2_ reacts with molten Li via Li-Mg alloying and sequential cleavage of TFSI⁻ anions, generating inorganic SEI species and Li-Mg alloy. X-ray photoelectron spectroscopy (XPS) analysis confirms key products. N 1*s* spectrum (Fig. 3b) shows Li_3_N (397.5 eV) from N–S bond cleavage; S 2*p* spectrum (Fig. 3c) reveals Li_2_S (161.2 eV), Li_2_S_2_ (162.8 eV), and Li_2_SO_4_ (168.4 eV), indicating stepwise S^6+^ reduction; F 1*s* spectrum (Fig. 3d) exhibits dominant LiF (684.8 eV); Mg 1*s* spectrum (Fig. 3e) verifies Li_3_Mg alloy (1304.8 eV) and oxidized Mg^2+^ (1303.2 eV). Figure 3f reveals the elementary steps of electrochemical TFSI^−^ breakdown involving the successive cleavage of N–S, S–C, C–F, and S=O bonds, ultimately forming small molecular weight Li salts such as LiF, Li_2_S_2_, Li_2_S, Li_3_N, etc. [20–22]. Figure S5 illustrates the surface SEM–energy-dispersive spectrometer (EDS) elemental mapping of Li@Mg(TFSI)_2_. The images show a homogeneous distribution of C-, F-, S-, N-, and Mg-related species derived from Mg(TFSI)_2_ reduction. The presence of these inorganic species on the outermost surface not only confirms the formation of a reduction product layer but also is expected to guide uniform Li deposition [23] and facilitate the formation of an inorganic-rich SEI. Cross-sectional images (Fig. S6), acquired using FIB-SEM, further show that the Mg(TFSI)_2_ reduction products exist as inorganic nanoparticles with diameters ranging from several tens of nanometers to ≈ 200 nm. These nanoparticles are distributed not only on the top surface but also in the near-surface region and inside the bulk Li metal layer, without evident settling or agglomeration. Such a homogeneous spatial distribution throughout the electrode is critical for constructing a genuinely effective passivating SEI.

The generated species are envisioned to synergistically regulate Li deposition behaviours [24]. LiF and Li_3_N of high ionic conductivity would enhance Li^+^ affinity and reduce charge transfer resistance [25], Li-Mg alloy (lithiophilic site) lowers nucleation barrier and promotes uniform Li plating [26], and meanwhile, the inorganics as LiF (70 GPa), Li_3_N (74 GPa), and Li_2_O (78 GPa) of high moduli reinforce SEI mechanical strength [27]. Deposited morphology of Li closely relates to the interactions between Li^+^ and plating substrate [28]. The bar chart in Fig. 3g compares Li^+^ adsorption energies (*E*ₐ_ds_) of diverse interphase components, revealing their critical role in guiding Li deposition behaviour. Lower* E*_ads_ indicates better lithiophilicity of substrate towards Li^+^, and Li nuclei sites are more easily formed [8]. Bare Li and Cu substrate exhibits high adsorption energies (− 0.009 and − 0.022 eV atom^−1^, respectively), indicating weak Li^+^ affinity that causes uneven nucleation and dendritic growth. In contrast, inorganic compounds derived from Mg(TFSI)_2_ decomposition, particularly Li_2_CO_3_ (− 0.251 eV atom^−1^), Li_2_S (− 0.119 eV atom^−1^), Li_3_Mg (− 0.041 eV atom^−1^), and Li_3_N (− 0.036 eV atom^−1^) display significantly lower *E*_ads_ values. These components introduce strong Li^+^-philic sites, reducing nucleation energy barrier and promoting uniform Li nucleation against dendritic Li growth.

**Fig. 4:**
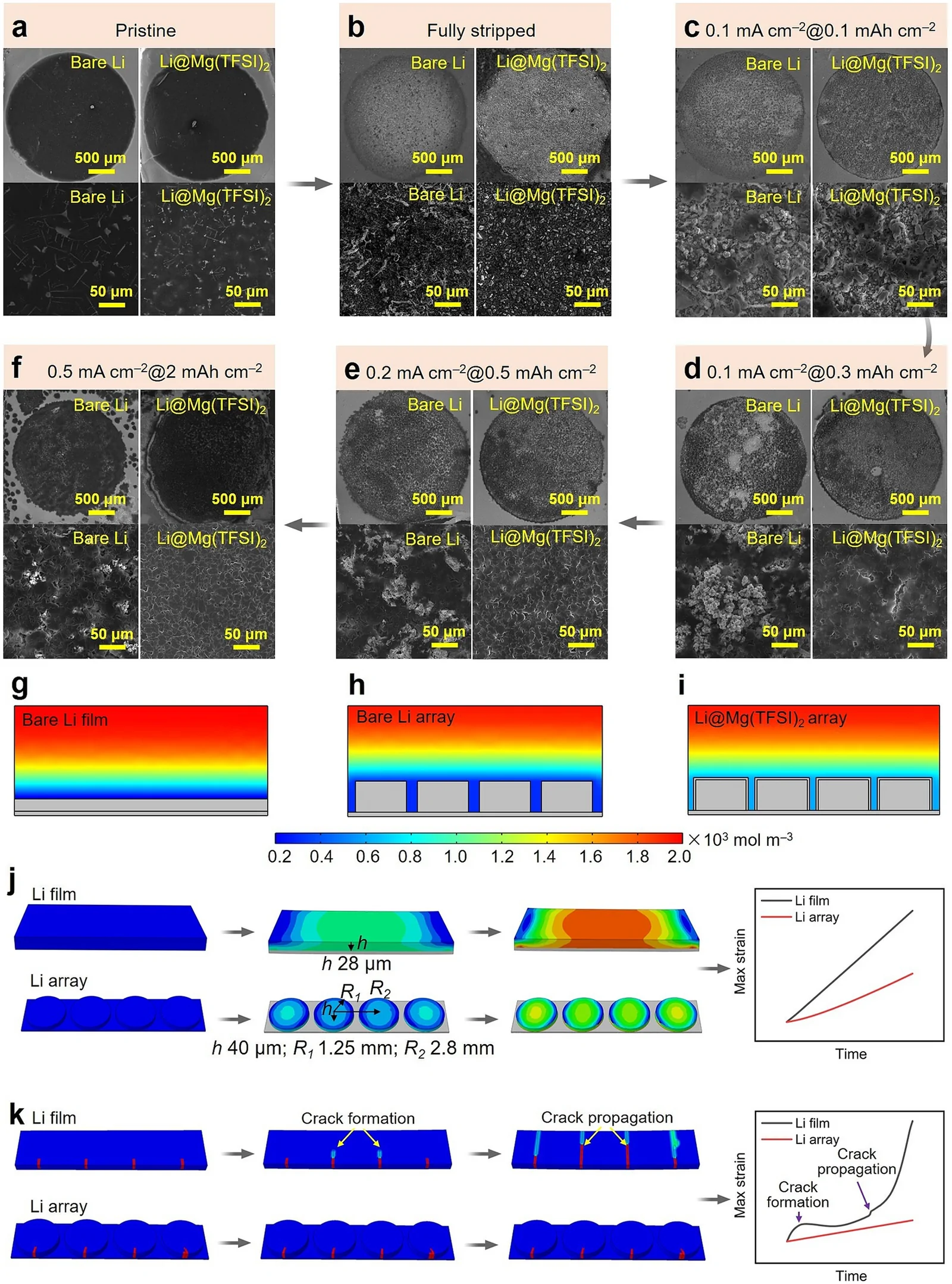
Li deposition morphology under different electrochemical states and FEA of Li^+^ concentration distribution and stress upon Li deposition. **a** SEM images of pristine bare Li and Li@Mg(TFSI)_2_. Initial state shows bare Li of rough surface *vs.* Li@Mg(TFSI)_2_ of smooth surface with embedded particles. **b** SEM images of bare Li and Li@Mg(TFSI)_2_ at fully stripped state. Fully stripped state suggests that bare Li leaves uneven Cu pits, while Li@Mg(TFSI)_2_ retains uniform inorganic particles on Cu. SEM images of bare Li and Li@Mg(TFSI)_2_ after Li depositions at **c** 0.1 mA cm^–2^@0.1 mAh cm^−2^, **d** 0.1 mA cm^–2^@0.3 mAh cm^−2^, **e** 0.2 mA cm^–2^@0.5 mAh cm^−2^ and **f** 0.5 mA cm^–2^@2 mAh cm^−2^, showing denser, more uniform deposits on Li@Mg(TFSI)_2_. Li^+^ concentration (mol m^−3^) distribution profiles of **g** bare Li film, **h** bare Li array, and **i** Li@Mg(TFSI)_2_ array. **j** Simulation of stress distribution in continuous Li film and Li array during Li deposition. Li array exhibits uniform stress distribution with lower maximum strain than the continuous film. **k** Crack propagation behaviour in Li film and Li array with identical pre-implanted microcracks. Li array suppresses crack growth and stress surge, while the continuous film shows rapid crack penetration and stress escalation

To directly visualize the impact of Mg(TFSI)_2_ doping on Li deposition behaviour, SEM characterizations under various electrochemical states were conducted (Fig. 4). As shown, the pristine Li@Mg(TFSI)_2_ anode (Fig. 4a, right) exhibits an overall smooth surface, with embedded inorganic particles (confirmed as LiF/Li_2_S/Li_3_N by XPS). After full stripping (Fig. 4b), bare Li leaves a rough Cu substrate with uneven pits and dendritic residues, while Li@Mg(TFSI)_2_ reveals a distribution of inorganic nanoparticles across the Cu surface. These particles act as lithiophilic nucleation sites for subsequent Li deposition. At low current deposition (0.1 mA cm^−2^@0.1 mAh cm^−2^, Fig. 4c), bare Li forms isolated dendritic clusters, whereas Li@Mg(TFSI)_2_ displays dense, granular Li nuclei. As deposition increases (0.1 mA cm^−2^@0.3 mAh cm^−2^, Fig. 4d), bare Li grows into mossy structures, while Li@Mg(TFSI)_2_ maintains a compact film with minimal porosity. Under higher current density (0.2 mA cm^–2^@0.5 mAh cm^–2^, Fig. 4e) and areal capacity (0.5 mA cm^–2^@2 mAh cm^–2^, Fig. 4f), Li@Mg(TFSI)_2_ sustains uniform, layer-like deposition, avoiding dendrite formation entirely. Be noted that lithium deposition behaviour on Li islands versus the Cu interspacing is governed by two factors: the lithiophilicity of the substrate and the amount of deposited Li. When the deposited Li capacity is relatively small (e.g., ≤ 2 mAh cm^−2^), Li^+^ ions preferentially nucleate and grow on the pre-existing Li islands rather than on the bare Cu interspacing. When the deposited capacity exceeds 2 mAh cm^−2^, reaching 5 mAh cm^−2^ or even 8 mAh cm^−2^ (Fig. S7a–c), Li begins to gradually deposit on the exposed Cu interspacing as well. The higher the deposition capacity, the more pronounced the deposition on Cu becomes. Importantly, compared with bare Li, Li@Mg(TFSI)_2_ exhibits stronger preferential deposition on the islands due to its more lithiophilic nature. Under 2 mAh cm^−2^, bare Li already shows noticeable Li deposition on the interspacing Cu, whereas Li@Mg(TFSI)_2_ shows almost none. Under higher capacities, Li@Mg(TFSI)_2_ consistently suppresses Li deposition on Cu more effectively than bare Li. This stark contrast confirms that Mg(TFSI)_2_-derived Li nanocomposite promotes homogeneous nucleation and suppresses dendritic growth, critical for stable cycling. This dual-modulation strategy involving both geometry and chemistry paves the way for high-performance Li metal anodes.

To further evaluate the electrochemical field distribution of the discrete Li microdisc architecture, finite element simulations (COMSOL Multiphysics) were conducted to map the Li^+^ concentration profiles near the electrode/electrolyte interface (Fig. 4g–i). Three configurations were compared: bare Li film, bare Li array, and Li@Mg(TFSI)_2_ array. Li@Mg(TFSI)_2_ array electrode exhibits the most homogeneous Li^+^ concentration across the entire surface, with negligible edge‑induced polarization. In contrast, bare Li film shows severe concentration polarization, while bare Li array displays moderate non‑uniformity at the disc boundaries. These results confirm that the combination of a discrete architecture and lithiophilic surface modification effectively homogenizes the ion flux, thereby promoting uniform Li deposition.

To address the critical issue of stress-induced mechanical failure in Li metal anodes [29], this work systematically compared the stress distribution and crack propagation behaviour between traditional continuous Li film and the proposed Li array configurations through multi-physical field simulations, as shown in Fig. 4j, k. Traditional continuous Li film suffers from severe non-uniform stress concentration during cycling. As Li deposition proceeds, localized stress at both the Li|Cu interface and Li bulk accumulates rapidly (Fig. 4j). This stress concentration arises from the continuous, unconstrained growth of Li, which generates large tensile/compressive forces that cannot be effectively dissipated [30]. In comparison, the Li array configuration enables uniform stress distribution. The discrete structure allows stress to be dispersed across multiple array elements. As a result, the maximum stress in Li array remains substantially lower than that of continuous Li film throughout deposition. This uniform stress state significantly enhances the mechanical stability of anode, mitigating the risk of stress-induced fracture or dendrite formation.

To further evaluate mechanical robustness, microcracks at identical positions in both continuous Li film and Li array are pre-implanted. During Li deposition, in continuous Li film, the pre-existing crack rapidly propagates and penetrates the entire film (Fig. 4k). This rapid propagation is driven by stress concentration at the crack tip, which amplifies as Li deposition continues. In contrast, Li array exhibits extraordinary resistance to crack growth as the pre-implanted crack shows negligible extension. The discrete Li dots act as “stress buffers”, preventing the crack tip from experiencing the high stress concentrations seen in the continuous film, which is consistent with the stress distribution trend in Fig. 4j (Videos S1 and S2). The Li array addresses two critical limitations of continuous Li film, i.e. dispersing stress via discrete structure and suppressing crack propagation by minimizing stress amplification at defect tips. Uniform stress distribution slows crack initiation, while the discrete structure prevents crack growth from becoming catastrophic. Together, they enable the Li array to maintain mechanical integrity over long cycles, a key requirement for practical Li metal batteries [31].

### Liquid-/Solid-State LMBs Building on Nanocomposite Arrays

Li@Mg(TFSI)_2_ array anode integrates mask-patterned Li microdiscs (structural innovation) with Mg(TFSI)_2_-derived inorganic nanodopants (compositional modification), envisioned to enable uniform Li deposition in both liquid- and solid-state batteries (Fig. S8). In liquid cells, the discrete array structure reduces local current density, while LiF/Li_2_S/Li_3_N particles from Mg(TFSI)_2_ decomposition provide lithiophilic nucleation sites. In solid-state cells, the array geometry enhances electrode/electrolyte contact, and the rigid SEI layer (rich in Li salts and Li-Mg alloy) buffers volume expansion, ensuring stable cycling. This strategy achieves dense, homogeneous Li plating/stripping manner regardless of electrolyte type.

**Fig. 5:**
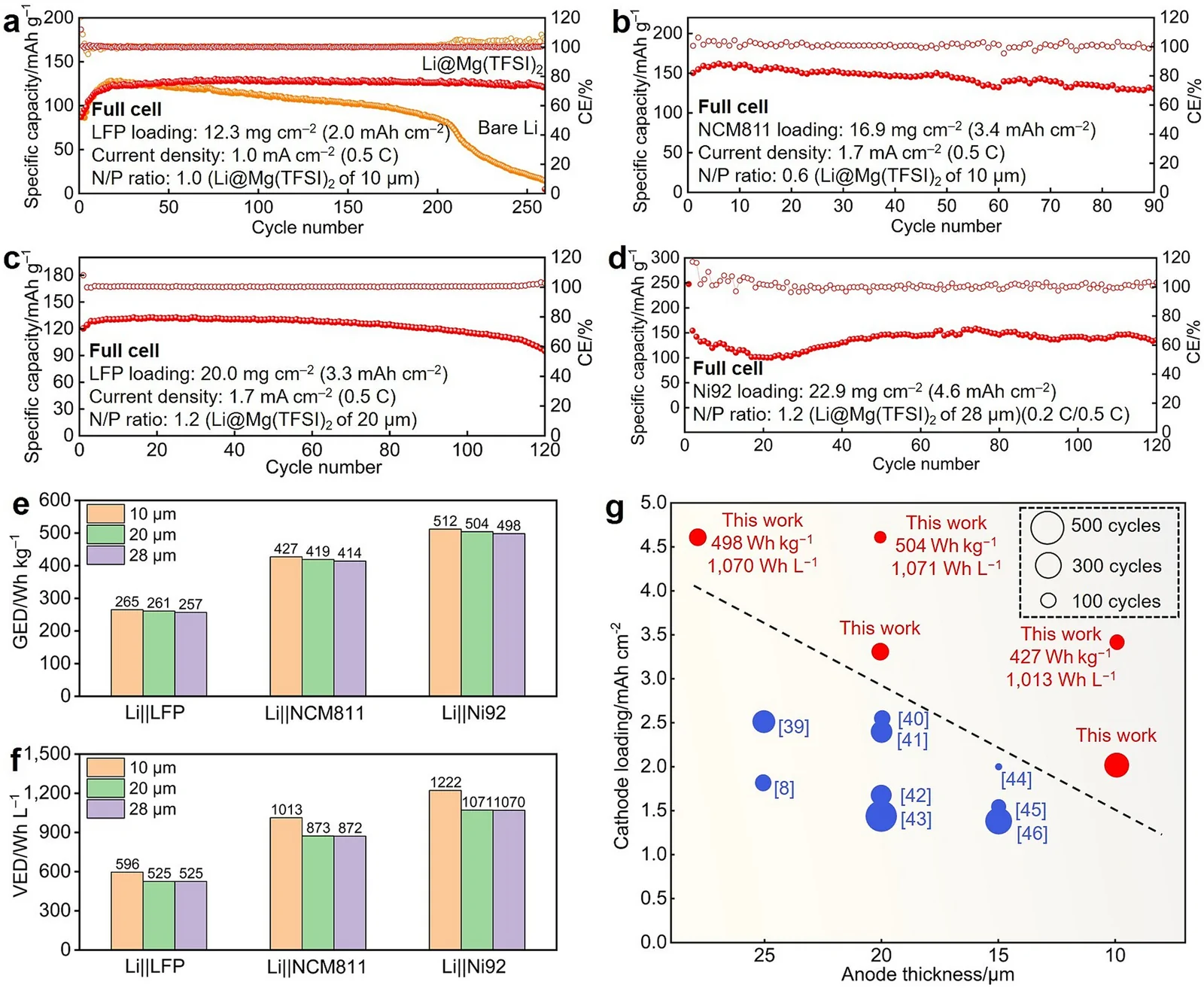
Electrochemical performance and energy density of Li@Mg(TFSI)_2_ array anodes based liquid LMBs. **a** Cycling performance of Li@Mg(TFSI)_2_ and bare Li arrays paired with LFP cathode (12.3 mg cm^−2^, N/P = 1.0). Cycling performance of full cells comprising Li@Mg(TFSI)_2_ anodes (equivalent 10 μm, 20 μm, and 28 μm) and high-loading cathodes. Cathodes including **b** NCM811 (16.9 mg cm^−2^), **c** LFP (20.0 mg cm^−2^), and **d** Ni92 (22.9 mg cm^−2^.) were used. Stack-level **e** GED and **f** VED of batteries consisting of Li@Mg(TFSI)_2_ anodes (equivalent 10 μm, 20 μm, and 28 μm) and high-loading cathodes (LFP, NCM811, and Ni92). **g** Plot of anode thickness vs. cathode loading of full cells, including the Li@Mg(TFSI)_2_-based cells and those from literature [8, 32–39]. Additional details and references are provided in Table S2

To evaluate the electrochemical performance of Li@Mg(TFSI)_2_ array anode, symmetric cells using equivalent 10 μm Li were assembled. Parallel symmetric cells (two each for bare Li and Li@Mg(TFSI)_2_) were tested at 1 mA cm⁻^2^@1 mAh cm^−2^ (Figs. S9 and S10). For Li@Mg(TFSI)_2_, both cells exhibit a stable overpotential of ≈ 40 mV at 500 h. For bare Li, the cells display a higher overpotential of ≈ 60 mV at 500 h and much shorter cycle life (530–670 vs. 800–1100 h for Li@Mg(TFSI)_2_). To address the critical trade-off between high energy density and long cycle life in liquid LMBs [10], Li@Mg(TFSI)_2_ array anode-based full cells were systematically investigated. The synergy of the design strategies enables exceptional cycling stability even under stringent conditions (high cathode loading and low negative to positive (N/P) ratios), while maximizing energy density through ultrathin anode configurations. Figure 5a compares the cycling performance of Li@Mg(TFSI)_2_ and bare Li arrays (both equivalent 10 μm) paired with LiFePO_4_ (LFP) cathode (12.3 mg cm^−2^, N/P = 1.0). The Li@Mg(TFSI)_2_||LFP cell maintains a high specific capacity (> 130 mAh g^−1^) with a stable *Coulombic* efficiency (CE > 99.8%) over 260 cycles, whereas the bare Li||LFP cell suffers from rapid capacity fade in 200 cycles.

To decouple the contributions of the array architecture and the Mg(TFSI)_2_-derived interphase chemistry, the cycling performances of bare Li film and Li@Mg(TFSI)_2_ film under identical conditions were performed (Fig. S11). Again, the Mg(TFSI)_2_-modified film exhibits markedly better capacity retention than the bare Li film, confirming the effectiveness of the chemical modification alone. However, Li@Mg(TFSI)_2_ array further outperforms the Li@Mg(TFSI)_2_ film, maintaining stable cycling over 260 cycles with negligible capacity decay, whereas the film counterpart begins to fade after ≈ 235 cycles. This indicates that the discrete array geometry by homogenizing the current distribution, buffering volume expansion, and mitigating mechanical stress synergizes with the Mg(TFSI)_2_-derived component modulation to deliver superior electrochemical stability. To further validate the versatility, Li@Mg(TFSI)_2_ anode was measured with a couple of high-loading cathodes under varying N/P ratios from 0.6 to 1.2 (Figs. 5b–d and S12). In particular with a ultra-high-loading and high-voltage LiNi_0.92_Co_0.03_Mn_0.05_O_2_ (Ni92) cathode (22.9 mg cm^−2^, 4.6 mAh cm^−2^), the full cells using either equivalent 20 μm (Fig. S12) or equivalent 28 μm (Fig. 5d) Li@Mg(TFSI)_2_ anode exhibit stable cycling up to 120 cycles. This performance underscores the anode’s ability to accommodate high-capacity cathodes under low N/P conditions, a critical requirement for practical high-energy batteries. Be noted that the specific capacity increase observed within the initial 20 cycles for the full cells is tentatively attributed to insufficient electrolyte wetting of the high‑loading cathodes at the early stage. Upon gradual electrolyte permeation, the accessible active material increases, resulting in a modest capacity rise before stabilization. This effect is amplified for higher‑loading Ni92 cathode (4.6 mAh cm^−2^) under asymmetric rate conditions (0.2 C charge, 0.5 C discharge).

Stack-level gravimetric and volumetric energy densities (GED and VED) were then calculated for batteries using Li@Mg(TFSI)_2_ anodes (equivalent 10 μm, 20 μm and 28 μm) and high-loading cathodes (LFP, LiNi_0.8_Co_0.1_Mn_0.1_O_2_ (NCM811) and Ni92) (Fig. 5e, f). GED of up to 512 Wh kg^−1^ and VED of 1222 Wh L^−1^ can be achieved. Even with thicker anodes (equivalent 20 μm and 28 μm), the GEDs (504 and 419 Wh kg^−1^, respectively) and VEDs (1071 and 873 Wh L^−1^, respectively) remain competitive, surpassing those of LFP and NCM811 systems pairing with Li films of 40–60 μm [40–45]. Be noted that the stack-level energy densities were calculated based on the application-relevant dimensions and proportions of the involved active/inactive materials (Table S1) [46]. Factors including N/P balancing, electrolyte excess, inactive cell components, volume change during (dis-)charge, etc. were considered.

A central trade-off in LMBs is that ultrathin anodes paired with high-areal-capacity cathodes boost energy density but often compromise cycle life [1]. The most extreme case is anode-free LMBs, which maximize device energy density by eliminating the Li anode entirely but generally suffer from severe capacity fade due to limited Li inventory [10]. Figure 5g contextualizes the Li@Mg(TFSI)_2_ anode’s performance against recent literature, plotting energy density against anode thickness and cathode loading. In literature, LMBs using thin Li anodes (≤ 25 μm) universally limit cathode areal capacities to ≤ 3 mAh cm^−2^ to balance energy density with modest cyclability. Higher cathode loads exacerbate Li depletion and dendrite growth, accelerating cell failure. However, Li@Mg(TFSI)_2_-based Li metal arrays present a promising strategy to mitigate this limitation. With equivalent 10–28 μm anodes, stable cycling up to 120 cycles is demonstrated using high-loading and high-voltage cathodes as NCM811 of 3.4 mAh cm^−2^ and Ni92 of 4.6 mAh cm^−2^. This yields exceptional energy densities (427–504 Wh kg^−1^, 1013–1071 Wh L^−1^) without compromising longevity.

**Fig. 6:**
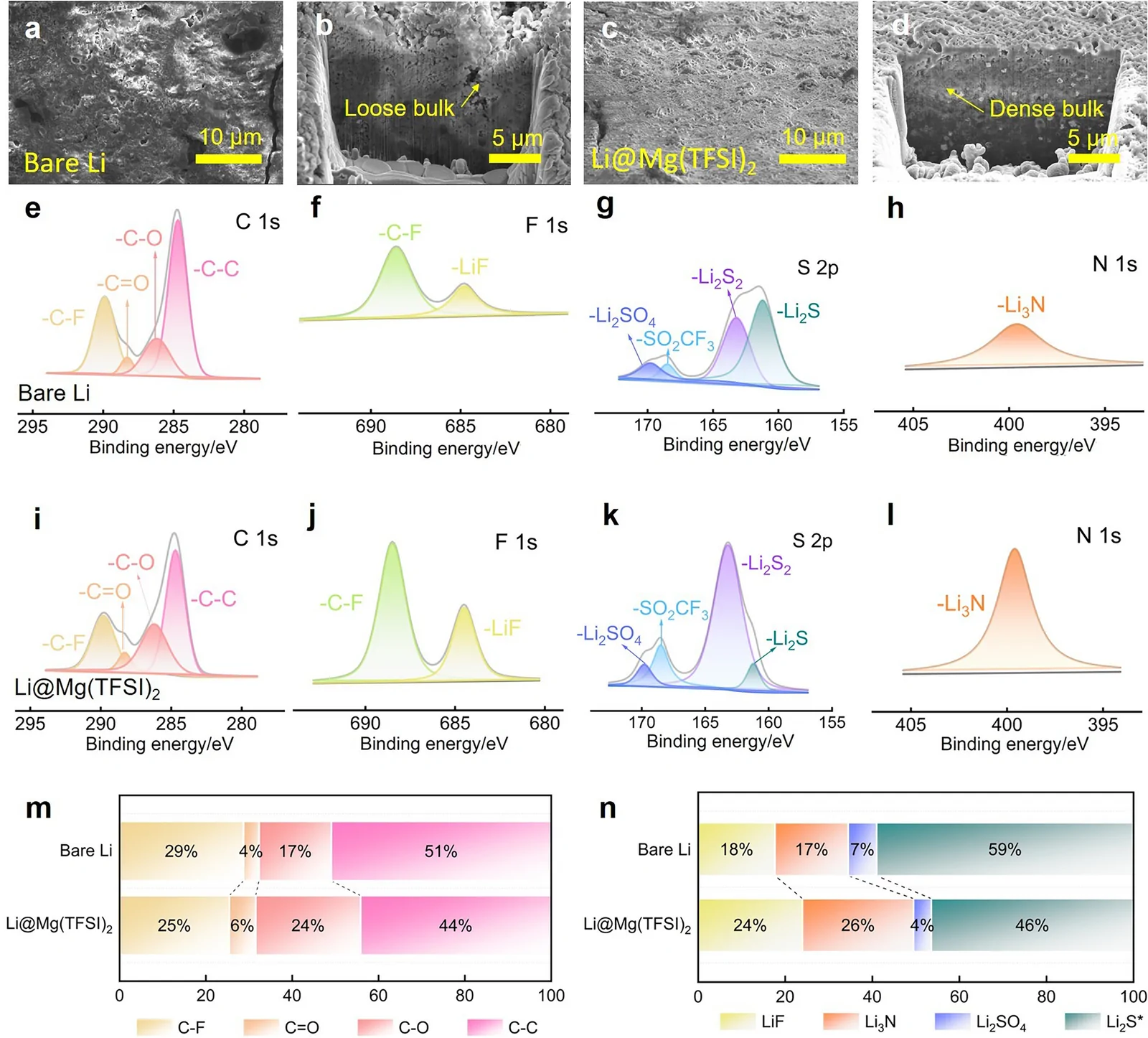
Microstructural and compositional analysis of Li deposition on bare Li vs. Li@Mg(TFSI)_2_ anodes. FIB-SEM images of **a, b** bare Li showing surface proliferation and loose structure *vs.* **c, d** Li@Mg(TFSI)_2_ with dense bulk structure. XPS spectra of cycled bare Li, including **e** C 1*s*, **f** F 1*s*, **g** S 2*p* and **h** N 1*s*. XPS spectra of cycled Li@Mg(TFSI)_2_, including **i** C 1*s*, **j** F 1*s*, **k** S 2*p*, and **l** N 1*s*. Elemental composition quantification of **m** organic C species and **n** inorganic SEI components of both cycled bare Li and Li@Mg(TFSI)_2_

Li deposition morphology and SEI dynamic evolution remain critical factors in determining the cycle life of LMBs [47]. The cross-sectional FIB-SEM images (Fig. 6a–d) and XPS analysis (Fig. 6e–l) reveal stark differences in Li deposition morphology and SEI composition between bare Li and Li@Mg(TFSI)_2_ arrays after cycling. For bare Li (Fig. 6a, b), the cycled anode exhibits severe surface proliferation with mossy/dendritic Li and porous bulk structure, attributed to unregulated Li growth and repeated SEI fracturing [48]. In contrast, Li@Mg(TFSI)_2_ (Fig. 6c, d) shows a dense, uniform bulk with minimal surface protrusions, confirming the structural stability induced by Mg(TFSI)_2_ doping. Moreover, bare Li’s SEI is dominated by organic species while Li@Mg(TFSI)_2_ exhibits a significant increase in inorganic components (Fig. 6m, n), indicating suppressed solvent decomposition [21]. This inorganic-enriched SEI would provide higher ionic conductivity and mechanical modulus, thus suppressing dendrite growth and volume fluctuation [49]. The synergy between structural array design and Mg(TFSI)_2_-derived SEI thus enables denser Li deposition and improved cycling stability.

### Unique Electrolyte/Electrode Interface Enables Stable Solid-State LMBs

**Fig. 7:**
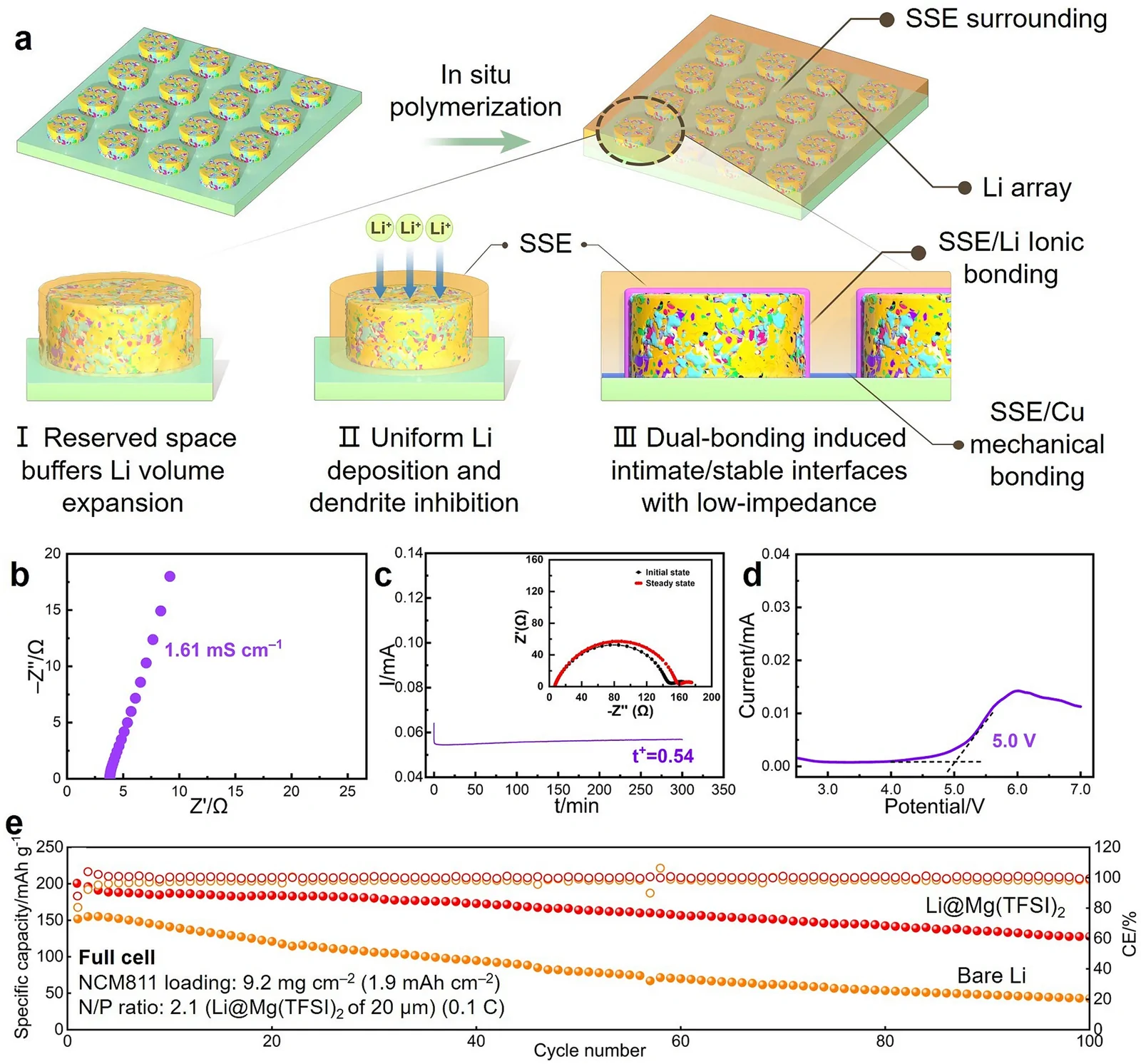
Electrochemical performance of Li@Mg(TFSI)_2_ array anode-based SSLMBs. **a** Structural design of Li@Mg(TFSI)_2_ anode in SSLMBs, illustrating volume expansion buffering, dendrite inhibition and dual-interface stabilization effects. **b** *Nyquist* plot of the in situ polymerized gel electrolyte, showing high ionic conductivity (1.61 mS cm^−1^). **c** Polarization curve of the in situ polymerized gel electrolyte confirms a high Li^+^ transference number (0.54). Inset graphs are the *Nyquist* plots before and after polarization. **d** Linear sweep voltammetry (LSV) curve of the in situ polymerized gel electrolyte indicates a wide electrochemical stability window (up to 5.0 V vs. Li/Li^+^). **e** Cycling performance of SSLMBs with high-loading NCM811 cathode, demonstrating more stable capacity retention and higher CE using the Li@Mg(TFSI)_2_ array anode than that of bare Li array anode

Figure 7 further demonstrates the performance of Li@Mg(TFSI)_2_ array anode in solid-state LMBs (SSLMBs), highlighting its compatibility with in situ polymerized solid-state electrolytes (SSEs) and validating its versatility beyond liquid systems. The Li@Mg(TFSI)_2_ array anode addresses key challenges in SSLMBs through rational design features (Fig. 7a). Firstly, reserved spaces between discrete Li array elements act as mechanical buffers, accommodating volume expansion during Li deposition and reducing stress accumulation, which are essential for maintaining structural integrity upon cycling. Secondly, compositional modification with Mg(TFSI)_2_ introduces Li compounds (e.g., LiF and Li_3_N) with high Li^+^ adsorption energy, which regulate uniform Li nucleation and growth, inhibiting dendrite formation that plagues traditional planar anodes [50]. Thirdly, the array configuration enables a dual-interface structure. One locates between SSE and Li array for efficient ionic conduction, and another between SSE and Cu current collector for robust mechanical bonding. This design provides multiple ionic transport paths while stabilizing the anode/SSE interface, a major bottleneck in SSLMBs.

An in situ polymerized gel electrolyte of favourable properties was used for SSLMBs in this work. The *Nyquist* plot in Fig. 7b evidences a high ionic conductivity of 1.61 mS cm^−1^ at room temperature, while the polarization curve in Fig. 7c confirms a high Li^+^ transference number of 0.54. The wide electrochemical stability window up to 5.0 V vs. Li/Li^+^ (Fig. 7d) indicates that this gel electrolyte is compatible with high-voltage cathodes. With such gel electrolyte, the Li@Mg(TFSI)_2_ array anode enables stable cycling in SSLMBs with high-loading cathodes, including NCM811 of 1.9 mAh cm^−2^ (Fig. 7e) and LCO of 2.0 mAh cm^−2^ (Fig. S13). The higher initial specific capacity of Li@Mg(TFSI)_2_ compared to bare Li (Fig. 7e) is attributed to its higher initial Coulombic efficiency and lower interfacial resistance (Fig. S14). These results suggest that the array anode’s structural and compositional advantages can be translated to practical performance in SSLMBs. Li@Mg(TFSI)_2_ array anode is validated as a versatile solution for both liquid- and solid-state cell scenarios, achieving the elusive balance of high energy density, long cycle life, and structural stability, which are the keys to advancing practical battery technologies.

## Conclusions

This study demonstrates a dual-modulation strategy through mask-assisted Li array fabrication and chemical modification to overcome critical barriers in ultrathin Li anodes. The mask-based process fundamentally solves the manufacturing challenge by avoiding molten Li’s poor wettability on copper substrate, a problem that traditionally causes high contact angles preventing the production of uniform sub-30-μm Li foils. The discrete array structure accommodates volume expansion during cycling, meanwhile reduces mechanical stress and inhibits dendrite formation. Complementing this, molten salt modification further optimizes interfacial chemistry through an inorganic-rich solid electrolyte interphase that minimizes Li consumption and electrolyte decomposition. These synergistic innovations enable ultrathin Li anodes (equivalent 10–28 μm) to deliver unprecedented electrochemical performance when paired with high-loading cathodes. Full cells achieve energy densities up to 504 Wh kg^−1^ and 1071 Wh L^−1^. By simultaneously resolving the fabrication difficulty and cycling instability that have long plagued ultrathin Li metal anodes, this work provides a technological solution for next-generation batteries. The demonstrated performance merits and process scalability position this approach as a promising pathway towards commercializing high-energy–density LMBs. Nevertheless, the Li array represents an entirely new form factor for Li metal anodes. Issues such as the exact influence of exposed Cu regions, the more complex electrode/electrolyte interfacial contact, and the evolution of local current density distribution remain to be systematically explored in future work. We hope this study inspires further efforts to understand and optimize such unconventional anode architectures.

## Supplementary Information

Below is the link to the electronic supplementary material.

Supplementary file1 (DOCX 5163 KB)

Supplementary file2 (MP4 3732 KB)

Supplementary file3 (AVI 3176 KB)

Supplementary file4 (AVI 3946 KB)
