# Supplementary material for: Screen-Printed Li Arrays with Reinforced Interphase as Ultrathin Anodes for High-Energy–Density Liquid- and Solid-State Batteries
Source: Nanomicro Lett. 2026 Aug 21;19:37. doi: 10.1007/s40820-026-02342-1 (PMC13498540; doi:10.1007/s40820-026-02342-1)
Supplement: Supplementary file 1 [file 40820_2026_2342_MOESM1_ESM.docx]

Supporting Information for

**Screen-Printed Li Arrays with Reinforced Interphase as Ultrathin Anodes for High-Energy-Density Liquid and Solid-State Batteries**

Wenting Liu^1, 2, ‡^, Zhenyao Wei^3, ‡^, De Ning^2, ‡^, Zhenhuan Lu^1,^ *, Fengping Zhang^2^, Xuhao Wang^2^, Binzhe Zhao^2^, Guohua Zhong^2^, Jie Zhang^2^, Yan Shao^2^, Ming Chen^2,^*, Man Wang^3^, Jun Wang^3,^ *, Chunlei Yang^2, 4^ and Wei Wu^2,^ *

^1^ Guangxi Key Laboratory of Electrochemical and Magneto-chemical Functional Materials, College of Chemistry and Bioengineering, Guilin University of Technology, Guilin 541004, P. R. China

^2^ Shenzhen Institutes of Advanced Technology, Chinese Academy of Sciences, Shenzhen 518055, P. R. China

^3^ Department of Materials Science and Engineering, School of Innovation and Entrepreneurship, Southern University of Science and Technology, Shenzhen 518055, P. R. China

^4^ Faculty of Materials Science and Energy Engineering, Shenzhen University of Advanced Technology, Shenzhen 518107, P. R. China

^‡^Wenting Liu, Zhenyao Wei, and De Ning have contributed equally to this work.

^*^Corresponding authors. E-mail: [zhenhuanlu@glut.edu.cn](mailto:zhenhuanlu@glut.edu.cn) (Zhenhuan Lu); [ming.chen2@siat.ac.cn](mailto:ming.chen2@siat.ac.cn) (Ming Chen); [wangj9@sustech.edu.cn](mailto:wangj9@sustech.edu.cn) (Jun Wang); [w.wu1@siat.ac.cn](mailto:w.wu1@siat.ac.cn) (Wei Wu)

**S1 Supporting Figures**

**Fig. S1** Optical image of as-prepared bare Li foil

**Fig. S2** Optical image of 20 μm-equivalent Li anode, dimension of ≈ 30 cm × 10 cm

**Fig. S3** Peel test of the screen‑printed Li microdisk array on Cu current collector. The Li microdisks remain firmly attached to the Cu substrate after peeling, demonstrating strong adhesion

**Fig. S4** Bending test of the screen‑printed Li microdisk array. After 200 bending cycles, the individual Li microdisks retain their original morphology and maintain intimate contact with the Cu substrate without visible detachment or mechanical damage

**Fig. S5** Surface SEM-EDS elemental mapping of Li@Mg(TFSI)_2_. **a** SEM image of the selected surface region. **b–f** Corresponding EDS maps of C, F, S, N, and Mg species, respectively. **g** Quantitative EDS spectrum of the Li@Mg(TFSI)_2_ surface, with the inset table summarizing the weight percentages of the detected elements

**Fig. S6** Cross-sectional FIB-SEM images of Li@Mg(TFSI)_2_ at different magnifications with a) 5 μm scale bar and b) 1 μm scale bar

**Fig. S7** SEM images of bare Li and Li@Mg(TFSI)_2_ after Li depositions at a) 0.5 mA cm^–2^@2 mAh cm^–2^, b) 0.5 mA cm^–2^@5 mAh cm^–2^, and c) 0.5 mA cm^–2^@8 mAh cm^–2^

**Fig. S8** Schematic of Li@Mg(TFSI)_2_ array anode in liquid/solid-state LMBs. Structural array design by discrete Li microdisks and compositional modification by Mg(TFSI)_2_-derived inorganic dopants synergistically enable uniform Li deposition, enhancing stability in both electrolyte systems

**Fig. S9** Voltage curves of two parallel symmetric cells using Li@Mg(TFSI)_2_ anodes at 1 mA cm⁻^2^@1 mAh cm⁻^2^

**Fig. S10** Voltage curves of two parallel symmetric cells using bare Li anodes at 1 mA cm⁻^2^@1 mAh cm⁻^2^

**Fig. S11** Cycling performance of bare Li film and Li@Mg(TFSI)_2_ film paired with LFP cathode (12.3 mg cm^−2^, N/P = 1.0) at 0.5 C

**Fig. S12** Cycling performance of full cell comprising Li@Mg(TFSI)_2_ anode of 20 μm and high-loading Ni92 cathode of 22.9 mg cm^−2^

**Fig. S13** Cycling performance of SSLMB with high-loading LCO cathode

**Fig. S14** a) First‑cycle voltage curves of Li@Mg(TFSI)_2_ and bare Li full cells (same cathode), showing a ≈ 10% higher initial Coulombic efficiency for the modified anode. b) Nyquist plots at fresh state and after the first cycle, demonstrating lower and decreasing interfacial resistance for Li@Mg(TFSI)_2_ versus higher and increasing resistance for bare Li.

**S2 Supporting Tables**

**Table S1** Parameters used in the energy density calculation at stack level conducted in this work

| Item | Parameters |
| --- | --- |
| Theoretical specific capacity of active materials (AM) (mAh g^−1^) | LFP: 170  NCM811: 279  Ni92: 279  Li metal: 3,862 |
| Practical specific capacity of AM (mAh g^−1^) | LFP: 150  NCM811: 200  Ni92: 210  Li metal: 3,862 |
| Mean discharge voltage (V) | LFP based LMB: 3.2  NCM811 based LMB: 3.7  Ni92 based LMB: 3.85 |
| Density (g cm^−3^) | LFP: 3.6  NCM811: 4.8  Ni92: 4.8  Li metal: 0.53 |
| Thickness of cathode (μm) | LFP of 20.0 mg cm^–2^: 84  NCM811 of 16.9 mg cm^–2^: 54  Ni92 of 22.9 mg cm^–2^: 68 |
| Electrolyte (g cm^−3^) | Density: 1.20 |
| Practical electrolyte excess with respect to pore volume | 15% |
| Separator (Celgard 2320) | Density: 0.95 g cm^−3^  Porosity: 39% |
| Binder and conductive additive | Binder: 1.80 g cm^−3^  Conductive additive: 2.25 g cm^−3^ |
| AM, binder, and conductive additive mass fraction | LFP cathode: 96:2:2  NCM811 cathode: 96:2:2  Ni92 cathode: 96:2:2  Li metal: 100:0:0 |
| Maximum volume change during discharge | LFP: −6.8%  NCM811: −5.9%  Ni92: −5.9%  Li metal: −100% |
| Electrode porosity | LFP cathode: 30%  NCM811 cathode: 30%  Ni92 cathode: 30%  Li metal: 0% |
| Current collectors | Al foil: 10 μm, 2.70 mg cm^−2^  Cu foil: 5 μm, 4.48 mg cm^−2^ |

**Table S2** Summary of thin Li foils fabricated using different strategies reported recently in comparison to this work

| Thickness of Li anode (μm) | | Cathode loading (mAh cm^−2^) | Cycling number | Publish year | | Ref. |
| --- | --- | --- | --- | --- | --- | --- |
| 25 | 2.5 | | 250 | 2023 | [S1] | |
| 25 | 1.8 | | 130 | 2024 | [S2] | |
| 20 | 2.5 | | 120 | 2025 | [S3] | |
| 20 | 2.45 | | 225 | 2023 | [S4] | |
| 20 | 1.6 | | 200 | 2022 | [S5] | |
| 20 | 1.5 | | 450 | 2025 | [S6] | |
| 15 | 2.0 | | 25 | 2023 | [S7] | |
| 15 | 1.5 | | 105 | 2023 | [S8] | |
| 15 | 1.5 | | 330 | 2023 | [S9] | |
| 28 | 4.6 | | 120 | - | **This work** | |
| 20 | 4.6 | | 60 | - | **This work** | |
| 20 | 3.3 | | 120 | - | **This work** | |
| 10 | 3.4 | | 90 | - | **This work** | |
| 10 | 2.0 | | 260 | - | **This work** | |

**Supplementary References**

[S1] Y. Hu, H. Li, Z. Chen, W. Cen, Q. Wang et al., Li-alloy texture creates in-built Li(110) epitaxy in a thin Li-metal anode allowing high depth-of-discharge cycling in carbonate electrolyte. Chem. Eng. J. **466**, 143084 (2023). <https://doi.org/10.1016/j.cej.2023.143084>
[S2] J. Cao, Y. Shi, A. Gao, G. Du, M. Dilxat et al., Hierarchical Li electrochemistry using alloy-type anode for high-energy-density Li metal batteries. Nat. Commun. **15**, 1354 (2024). <https://doi.org/10.1038/s41467-024-45613-4>
[S3] G. Zhang, T. Zhang, Y. Liu, Q. Wang, R. He et al., Molecular design of asymmetric difluorinated ether electrolytes for stable operation of high-voltage lithium metal batteries. Angew. Chem. Int. Ed. **64(38)**, 506056 (2025). <https://doi.org/10.1002/anie.202506056>
[S4] J. Xing, T. Chen, L. Yi, Z. Wang, Z. Song et al., Endowing Cu foil self-wettable in molten lithium: a roll-to-roll wet coating strategy to fabricate high-performance ultrathin lithium metal anodes. Energy Storage Mater. **63**, 103067 (2023). <https://doi.org/10.1016/j.ensm.2023.103067>
[S5] Y. Zhao, T. Zhou, L.P.H. Jeurgens, X. Kong, J.W. Choi et al., Electrolyte engineering for highly inorganic solid electrolyte interphase in high-performance lithium metal batteries. Chem **9(3)**, 682–697 (2023).  <https://doi.org/10.1016/j.chempr.2022.12.005>
[S6] L. Deng, Y. Liu, H. Qi, Y. Yang, Z. Wang et al., A nanoengineered lithium-hosting carbon/zinc oxide composite electrode material for efficient non-aqueous lithium metal batteries. Nat. Nanotechnol. **20**, 1439–1448 (2025). <https://doi.org/10.1038/s41565-025-01983-4>
[S7] J. Gao, C. Chen, Q. Dong, J. Dai, Y. Yao et al., Stamping flexible Li alloy anodes. Adv. Mater. **33(11)**, 2005305 (2021). <https://doi.org/10.1002/adma.202005305>
[S8] L. Fu, X. Wang, B. Zhang, Z. Chen, Y. Li et al., A Li_3_P nanoparticle dispersion strengthened ultrathin Li metal electrode for high energy density rechargeable batteries. Nano Res. **17(5)**, 4031–4038 (2024). <https://doi.org/10.1007/s12274-023-6275-9>
[S9] S. Huang, Z. Wu, B. Johannessen, K. Long, P. Qing et al., Interfacial friction enabling ≤ 20 μm thin free-standing lithium strips for lithium metal batteries. Nat. Commun. **14**, 5678 (2023). <https://doi.org/10.1038/s41467-023-41514-0>
